# The impact of neuroscience on society: cognitive enhancement in neuropsychiatric disorders and in healthy people

**DOI:** 10.1098/rstb.2014.0214

**Published:** 2015-09-19

**Authors:** Barbara J. Sahakian, Annette B. Bruhl, Jennifer Cook, Clare Killikelly, George Savulich, Thomas Piercy, Sepehr Hafizi, Jesus Perez, Emilio Fernandez-Egea, John Suckling, Peter B. Jones

**Affiliations:** 1 Department of Psychiatry, University of Cambridge, Herchel Smith Building for Brain and Mind Sciences, Forvie Site, Robinson Way, Cambridge CB2 0SZ, UK; 2 MRC/Wellcome Trust Behavioural and Clinical Neuroscience Institute, University of Cambridge, Cambridge CB2 3EB, UK; 3 Department of Psychiatry, Psychotherapy and Psychosomatics, Psychiatric Hospital, University of Zurich, Lenggstrasse 31, 8032 Zurich, Switzerland; 4 CAMEO North Team, 3 Thorpe Road, Peterborough PE3 6AN, UK; 5 CAMEO South Team, Block 7, Ida Darwin Site, Fulbourn, Cambridge CB21 5EE, UK

**Keywords:** cognitive enhancers, neuroethics, cognitive training, schizophrenia, game, smart drugs

## Abstract

In addition to causing distress and disability to the individual, neuropsychiatric disorders are also extremely expensive to society and governments. These disorders are both common and debilitating and impact on cognition, functionality and wellbeing. Cognitive enhancing drugs, such as cholinesterase inhibitors and methylphenidate, are used to treat cognitive dysfunction in Alzheimer's disease and attention deficit hyperactivity disorder, respectively. Other cognitive enhancers include specific computerized cognitive training and devices. An example of a novel form of cognitive enhancement using the technological advancement of a game on an iPad that also acts to increase motivation is presented. Cognitive enhancing drugs, such as methylphenidate and modafinil, which were developed as treatments, are increasingly being used by healthy people. Modafinil not only affects ‘cold’ cognition, but also improves ‘hot’ cognition, such as emotion recognition and task-related motivation. The lifestyle use of ‘smart drugs' raises both safety concerns as well as ethical issues, including coercion and increasing disparity in society. As a society, we need to consider which forms of cognitive enhancement (e.g. pharmacological, exercise, lifelong learning) are acceptable and for which groups (e.g. military, doctors) under what conditions (e.g. war, shift work) and by what methods we would wish to improve and flourish.

## 1. Cognitive enhancement in neuropsychiatric disorders

In addition to the increasing prescription use of cognitive enhancers, there is also a growing lifestyle use by healthy people [1]. The evidence for this trend comes mostly from increasing sales numbers, in addition to purchases via the internet and sporadic seizures such as reported in a recent newspaper article about a £200 000 smart drugs seizure [2]. However, solid longitudinal data on cognitive enhancing drug use by healthy people using rigorous survey methodology are needed. There is also a lack of data on the dangers of these drugs. For example, in the case of the racetam class, piracetam is relatively well studied. However, the newer racetams being sold via the internet are far more potent and indeed, some drugs being sold have no published clinical trials on their safety and efficacy. For these and other neuroethical reasons, including justice, the Presidential Commission for the Study of Bioethical Issues has reported on cognitive enhancing drugs as one of the top four priorities in their recent Gray Matters report [3].

To get a better understanding of potential effects, benefits and also potential dangers of so-called smart drugs, we first need to define this group of drugs. From a scientific point of view, the definition of ‘enhancing’ is difficult: does it refer to an improvement of a function relative to its previous level or beyond a particular point [4]? This might seem relatively easy to define in patients with clear cognitive deficits such as in chronic schizophrenia or stroke, but might prove more difficult in people without a medical diagnosis but subjective deficits or in healthy people with the wish to improve their cognitive performance. For example, a healthy person who is sleep-deprived or jet lagged may want to counteract that state with cognitive enhancing drugs. Would that be considered restoration or enhancement? Similarly, an elderly person who is still working may wish to perform as they did when they were in their twenties. Again, if they use cognitive enhancing drugs to obtain their previous optimal state, would that be considered restoration or enhancement? From a pharmacological perspective, drugs with a supposed cognitive enhancing effect can be found in a variety of classes with a variety of pharmacological targets ranging from acetylcholine to serotonin, glutamate, noradrenaline and dopamine, plus drugs where the mechanism by which they exert their cognitive enhancing effects is still not fully understood. The aim of this review is to summarize studies on cognitive enhancers in patients, including a new study of our group on a non-pharmacological method of enhancing motivation and cognition. The review also presents research studies on healthy volunteers that use drugs as tools to understand the neuromodulation by neurotransmitters of cognitive processes, including attention, memory, planning and problem solving. In addition, the article discusses the increasing use of ‘smart drugs' by healthy people as well as the use of neuromodulating devices in both disorders and health. Finally, we consider the implications for society and future research directions.

Neuropsychiatric disorders are disorders of cognition. Cognitive manifestations of neuropsychiatric disorders include disturbances in the regulation of attention (attentional biases), learning (aberrant learning), and in top–down regulation by the prefrontal cortex [5]. Furthermore, impairments of memory and executive function have been found in many neuropsychiatric disorders [6–12]. Many neuropsychiatric disorders have a neurodevelopmental origin and an onset or prodromal stage in childhood [13], and mental disorders affect young people disproportionally, with 75% of mental illnesses beginning before the age of 24 years. This is the period during which the brain is undergoing major developmental processes, particularly in the prefrontal cortex [14], which is why it is not that surprising that environmental influences, such as stress or substance abuse, may have especially profound effects on prefrontal functions [5].

Neuropsychiatric disorders affect the functionality and wellbeing of individuals and pose an economic burden for society [15]. These disorders are not only costly with respect to treatment and other services related to disorders, including special accommodation, social services and informal care, but even more so when considering the indirect costs, such as lost income and productivity owing to not finishing school or reaching only low grades, inability to train for a job, lower academic achievement, absence from work or early retirement [16]. Currently, treatment for mental disorders, both psychologically and pharmacologically, targets the obvious symptoms of the disorders, but rarely the cognitive impairments (except for some treatments for Alzheimer's disease and attention deficit hyperactivity disorder (ADHD)). These cognitive symptoms often persist even after remission of the more acute symptoms of, for example depression or psychosis [17–20], and they have a great impact on the indirect costs and loss of productivity, quality of life and wellbeing [21–24]. Therefore, ‘smart drugs' or cognitive enhancing drugs are needed to improve cognitive problems in patients with neuropsychiatric disorders. Improving functionality of patients suffering from neuropsychiatric disorders will lead to a reduction of the costs of these disorders for society and governments in the range of about £90 billion in the UK [25].

A wide variety of interventions that can enhance cognitive performance in mental disorders have been identified [26]. Some of them are behavioural interventions, such as exercise, meditation, sleep hygiene or cognitive stimulation (e.g. cognitive training, CT). Others involve the application of exogenous agents such as cognitive enhancing drugs or nutritional supplements with supposedly positive effects on cognitive performance and mood [27]. A last group of interventions targets the brain physically by applying electromagnetic fields to the surface of the skull, e.g. transcranial magnetic stimulation (TMS) or transcranial direct current stimulation (tDCS). All of these interventions aim to improve neural circuits in a neuroplastic way and to improve cognitive functions (including socio-affective functions).

### (a) Alzheimer's disease

Alzheimer's disease, a neurodegenerative disorder characterized by a progressive decline in cognitive and everyday functioning, was one of the first disorders where acetylcholinesterase inhibitors were developed and are now approved and widely used specifically to improve the impaired cognitive function or to reduce the cognitive decline (e.g. [28]). In Alzheimer's disease and other dementias the most obvious symptoms from the beginning are cognitive deficits, one of the earliest impairments being in episodic memory [29,30]. The current guidelines on pharmacological treatment in Alzheimer's dementia consistently recommend early assessment and treatment with cholinesterase inhibiting drugs such as donepezil, galantamine and rivastigmine beginning early and continuing as long as tolerated [31–36]. The NMDA receptor antagonist memantine is also generally used in later stages of Alzheimer's disease. Evidence suggests that these drugs can also be effective even in severe disease states [31]. There are currently 820 000 people with dementia in the UK, with one new case every 14 minutes in England and Wales, and the cost of dementia to the UK economy is £23 billion per year, which is more than cancer, heart disease and stroke [32]. The number of people placed in institutions is expected to rise from 224 000 in 1998 to 365 000 in 2031. However, early assessment and early effective treatment with drugs aimed at improving cognitive function have been shown to reduce overall direct costs by more than £3600 per patient, with another £4150 in indirect cost savings [33], parallel to an improvement of quality of life for the patient and the carers.

### (b) Parkinson's disease

Parkinson's disease (PD) is a chronic progressive neurodegenerative disorder typically characterized by motor deficits, but a majority of patients also suffer from non-motor symptoms that impact on their quality of life [34]. Non-motor symptoms in PD include psychiatric symptoms such as mood and affect symptoms and psychosis, cognitive dysfunction, fatigue and dementia, and autonomic dysfunction. More than 125 000 people are affected by PD in the UK [35], causing total costs of about £1.9 billion per year [16]. In parallel to the gradual loss of function, the need for care increases, placing a high burden on families and society as a whole. With the increasing age of the population, the number of persons affected by PD is expected to rise. The non-motor symptoms contribute considerably to the burden for carers and the reduced quality of life in PD patients, as well as nursing home placement [36]. Cognitive enhancers have been investigated in order to treat cognitive dysfunction and fatigue, as well as other dementia symptoms [37]. Studies have tried to improve fatigue using methylphenidate [38] and modafinil [39,40], both with only weak effects. Impulsivity can on the one hand be a symptom of PD itself, and on the other hand be evoked by treatment with dopamine agonists in vulnerable patients (review: [41]). One study using amantadine, an NMDA receptor antagonist with additional effects on dopamine, norepinephrine and acetylcholine, found weak effects on clinical measures of gambling in PD [42]. Another study in our laboratory used a single dose of the noradrenergic reuptake inhibitor atomoxetine [43], which also has antagonistic effects on the NMDA receptor [44]. In a double blind placebo-controlled crossover design, PD patients showed a trend to an improved reaction time and accuracy on the stop signal reaction test (SSRT), reduced risk-taking behaviour and increased deliberation time in the Cambridge Gamble Task [45]. There was also a positive correlation between atomoxetine plasma concentration and planning performance on the One Touch Stockings of Cambridge task (www.camcog.com). On the neural level, this effect was associated with an increased activation in inhibition-related brain areas, including the right inferior frontal gyrus, and fronto-striatal connectivity [46]. These promising proof-of-concept studies support further testing of atomoxetine treatment in impulsive–compulsive PD in clinical studies with longer-term treatment. Regarding dementia and other specific cognitive symptoms, anticholinergic drugs, such as donepezil and rivastigmine, are considered as efficacious but have rather weak effects, whereas the NMDA receptor antagonist memantine shows no clear positive effect (review [37]). One study showed improved global cognition with atomoxetine in PD [47], parallel to another study that showed improved executive functioning on two clinical scales of executive function [48].

### (c) Attention deficit hyperactivity disorder

Attention deficit hyperactivity disorder is a highly heritable disorder (in children about 75% heritable; meta-analysis [49], review [50]) with a 12-month prevalence of 5–6% in children [51] and about 4% in adults [52]. If ADHD is not treated effectively, the prognosis is complicated by psychiatric comorbidities such as anxiety, mood and substance-use disorders and impairments in basic, social and productive role functioning [52]. Longitudinal studies indicate that ADHD is associated with poorer long-term outcomes, including increased educational dropout, job dismissal, criminal activities, substance abuse, other mental illnesses and increased accident rates [53], which can be reduced by treatment [54], but only partially when looking at academic or occupational outcome [53,55]. While methylphenidate (Ritalin^®^) is an effective treatment for about 60–70% of people with ADHD, some people do not respond to treatment or cannot take the treatment owing to side effects [56]. In addition, methylphenidate is a stimulant drug and therefore has some abuse potential [57]. For these reasons, it is important that novel, effective and safe treatments for ADHD are developed. The annual excess cost of ADHD in the USA in 2000 was estimated to be $31.6 billion [58].

Working memory is one of the cognitive domains impaired in ADHD in childhood [59,60] and adulthood [61]. Methylphenidate, acting primarily by increasing the synaptic concentration of dopamine and noradrenaline by blocking their re-uptake, improves spatial working memory performance in adult patients with ADHD [62] and in healthy volunteers [63]. On the neural level, the main effect of methylphenidate is an increase in efficiency of the networks involved in working memory [64]. The main locus of action in both patients with ADHD and in healthy volunteers is the striatum [65]. However, this mechanism seems not to be different between patients with ADHD and healthy volunteers, who do not differ with respect to their dopamine D2/3 receptor capacity at baseline [65]. Furthermore, methylphenidate improved and normalized the stop signal reaction time in boys with ADHD [66]. A recent meta-analysis of methylphenidate effects in children and adolescents confirmed this positive effect on response inhibition, as well as on the increased reaction time variability [67]. In parallel, modafinil improved response inhibition in ADHD [68]. Atomoxetine also improved response inhibition in adult patients with ADHD [69], but did not affect sustained attention and spatial working memory.

### (d) Schizophrenia

Schizophrenia is a paradigmatic disorder where cognitive deficits on the one hand are a core domain [8,70,71] and on the other hand are known to be an important determinant of functional outcome through their impact on the level of self-care and utilization of hospital services [72–76]. Overall, summing-up direct and indirect costs, schizophrenia is estimated to cost £ 13.1 billion per year in total in the UK [16,77]. However, even small improvements in cognitive functions could help patients make the transition to independent living and working [26,74] and could therefore reduce direct and indirect costs markedly, besides improving the wellbeing and health of patients. Cognitive deficits are typically found in memory, cognitive flexibility and visuospatial learning [78]. In patients with a first psychotic episode, modafinil improved working memory [71] and also emotion recognition [79]. In patients with manifest schizophrenia, modafinil improved cognitive flexibility, measured with the intra-extradimensional attentional set shifting task [80]. Other studies showed improved motor activity [81] and improved clinical condition and self-reported quality of life, as well as improved cognitive performance on a subtest of the Wechsler adult intelligence scale [82]. On the neurobiological level, modafinil increased dorsolateral prefrontal cortex (DLPFC) activity, particularly in schizophrenia patients with cognitive dysfunction [83]. Acetylcholinesterase inhibitors, such as donepezil and rivastigmine, have shown no or only weak effects in schizophrenia (reviewed in [84]). Galantamine acts as cholinesterase inhibitor, but also on nicotinic acetylcholine receptors parallel to nicotinic agonists such as xanomeline and DMXB-A and nicotine. Galantamine improved certain cognitive functions in schizophrenia, but further research studies are required [84,85].

There is a great need to develop novel, more effective cognitive-enhancing drugs to improve cognition, functionality and wellbeing in patients. However, regrettably, many pharmaceutical companies have left the field of drug development in neuropsychiatric disorders, such as ADHD and schizophrenia, although some remain in pursuing neuroprotective treatments for neurodegenerative conditions such as Alzheimer's disease [86]. For this and other reasons, including the explosion of technological advances underused in mental healthcare, there is an unmet need to develop cognitive enhancement techniques, such as CT, which can be used on a much larger scale [5,87]. As an example of these possibilities that represent the futurescoping of cognitive enhancement, we present data on CT of memory using an iPad game in patients with schizophrenia. As apathy, anhedonia and negative symptoms are typical of chronic neuropsychiatric disorders, the game training has the added advantage of increasing motivation. This latter feature of CT games is particularly important as patients actively enjoy participating, in contrast to the traditional CT, which may seem tedious or boring.

### (e) A study on computer-game-based CT in schizophrenia

Cognitive remediation therapies for schizophrenia result in improvements in cognition, symptoms and psychosocial functioning, particularly when combined with neuropsychiatric rehabilitation (meta-analyses: e.g. adjunctive social skills training, vocational training; [88,89]). Of particular interest, recent computer-assisted cognitive rehabilitation and training studies have successfully shown improvements, not only in cognitive functioning, but also in negative symptoms [90], reality monitoring [91], social cognition [92] and employment [93,94] and functional [95] outcomes in schizophrenia. We therefore conducted a study to test the promise of computer-based rehabilitation programmes for schizophrenia and further suggest their potential for helping patients achieve better functional outcomes (e.g. work, independent living), one critical component of rehabilitation. A recent systematic review of rehabilitation studies for schizophrenia concluded that a range of cognitive domains could be improved using computerized practice, suggesting that episodic memory difficulties may be one cognitive dysfunction amenable to computer-based CT [96]. Therefore, we focused on episodic memory for training, because we had previously demonstrated that it was related to functional outcome in patients with first episode psychosis, and because we had a strong understanding of the underlying neurocircuitry involved [29,97]. However, CT packages typically require supervision on standard psychological paradigms. Sessions are often long and repetitive; dropout rates can be high and cost efficiency is limited by the requirement for constant supervision. For example, Wykes *et al*. [88] note that the dropout rate for 12 of the studies included in their meta-analysis of cognitive remediation for schizophrenia was higher than 15%. Recent neuroscience and mental health policy reports suggest that the use of neurotechnologies, such as video-game training, is a potential technique for enhancing cognitive performance in schizophrenia [86,87,98–100]. Computer-based CT taking the form of a neurotechnology (i.e. ‘brain training’ software), for example, has already been shown to improve verbal learning/memory and cognitive control in individuals with schizophrenia. Importantly, improved cognition was associated with better functioning 6 months after completing the training intervention [101]. Use of a computer game as a means of cognitive remediation has also been shown to improve negative symptoms and executive function in a small number of individuals with first episode psychosis [102]. Furthermore, video game playing has been associated with structural neural changes, including a significant grey matter increase in the hippocampal formation in healthy individuals [103]. Computer games that are custom-made to be enjoyable, attention-grabbing and easily accessible may thus comprise an appealing treatment option for patients and a cost effective option for health services.

In this study, we developed a novel memory game for delivering CT of episodic memory using a hand-held portable iPad. This game embedded a paired associative learning (PAL) task into a narrative, which allowed for the selection of characters, rewarded progress, provided feedback and used visually appealing displays and stimulating music to keep users engaged and motivated. The Cambridge neuropsychological test automated battery (CANTAB) PAL task is a sensitive measure of episodic memory that has been shown to engage the hippocampal formation [29]. We aimed to test the effectiveness of 8 h of CT (i.e. gameplay) on memory and daily functioning in individuals with schizophrenia. We also measured participants' level of enjoyment and motivation to continue gameplay to determine whether a wider application to other patient groups would be feasible for future larger studies.

#### (i) Methods

Twenty-two participants were recruited from public health services within the Cambridgeshire and Peterborough Foundation Trust (CPFT), including the Cambridge Assessment and Management of Early Outcomes service (CAMEO; www.cameo.nhs.uk) and the Rehabilitation and Recovery Service, as well as analogous services in other parts of the UK (see table 1 for demographic information). Inclusion criteria were patients with a clinical diagnosis of schizophrenia as determined by the SCID-P16 [104] or DSM-5 [105] or a related condition (e.g. schizoaffective disorder, schizophreniform disorder). All patients were clinically stable and treated with anti-psychotic medication during the course of the study, except for five patients (one pregnant; four recently discharged from specialist clinical services). Participants were excluded from the study if they had a current or past neurological condition or disorder, including epilepsy or head trauma with loss of consciousness. Only adults (age > 16) were recruited. Participants were excluded if they did not have normal or corrected-to-normal vision (6/9 or better). This study received full ethical approval by the Cambridge Central Research Ethics Committee (12/EE/0018). All participants gave written consent prior to enrolling. To reduce placebo effects, participants were not informed at the time of testing that the study concerned CT of episodic memory, but rather were told that the aim was to investigate the effect of iPad use on psychological processes. Participants were randomly assigned to either the CT or treatment as usual (TAU) groups. All participants first completed the National Adult Reading Test (NART [106]) and the Brief Psychiatric Ratings Scale (BPRS, [107]) as measures of intelligence and psychiatric symptoms, respectively. Participants in the CT group played the memory game for a total of 8 h over a 4-week period; participants in the TAU group continued their TAU. All participants completed the CANTAB PAL and the Global Assessment of Functioning (GAF, [108]) at both baseline and outcome time points. The outcome testing session took place exactly 4 weeks after the baseline testing session. The CANTAB PAL is an episodic memory task comprised of one-pattern, two-patterns, three-patterns, six-patterns and eight-patterns stages. The GAF was used to numerically rate participants' social, occupational and psychological function. The BPRS results in a sum score, and we additionally calculated a negative symptom score by averaging the BPRS subscales Emotional Withdrawal, Motor Retardation and Blunted Affect [109]. Statistical analyses were done using SPSS 20. We used *t*-tests, Mann–Whitney tests, ANCOVAs and bivariate correlations as appropriate, depending on the normality of the distribution of the data. As the PAL total errors and total trials were not normally distributed, the values were square root transformed for statistical analyses. However, for better comparison with other studies, table 2 reports the absolute scores.

#### (ii) Memory game development

To ensure that our training programme took the form of a memory game rather than a standard psychological test, we employed a professional game designer who was briefed to develop an entertaining game that incorporated a learning and episodic memory task into a narrative. Once a prototype had been developed—using six focus groups, each with two individuals with a diagnosis of schizophrenia—the game was further developed until focus group participants agreed that the game was fun, attention-grabbing, motivating and easy to understand. The game required the patient to remember correctly the location of patterns in space. The game started in an easy stage at the beginning but increased in difficulty as the participant succeeded, using a ‘three up one down’ adaptive staircase, with the highest level requiring participants to memorize the locations of 20 runes. The memory game employed a number of strategies to maintain a high level of motivation to continue gameplay: the episodic memory task was woven into a narrative with individualized aspects (choice and name of the character), appealing design and stimulating music; the game rewarded progress with additional in-game activities to provide the user a sense of progression independent of the CT process while minimizing failure. After each hour of gameplay (hours 1–8), participants in the CT group completed a visual analogue scale to rate their enjoyment and motivation to continue playing the game (scores ranged from 0 to 100, with higher scores indicating enhanced enjoyment/willingness to continue).

#### (iii) Results

**Figure 1.**
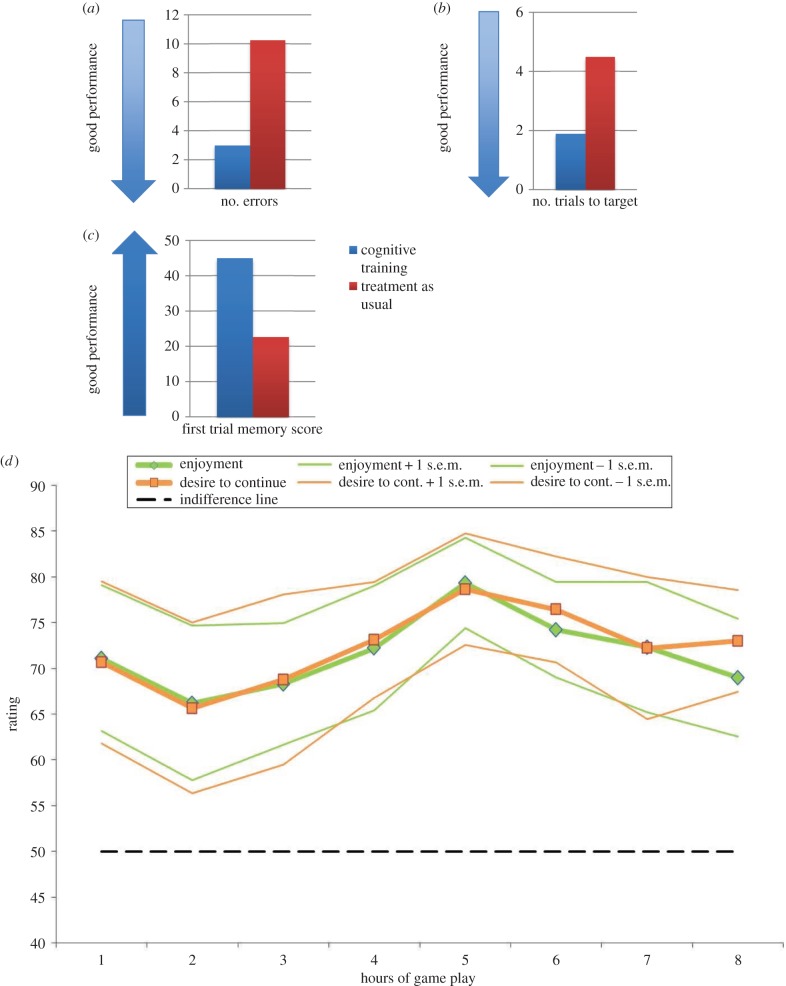
Memory game training in schizophrenia: the cognitive training (CT) group made fewer errors (*a*), needed fewer trials to target (*b*) and correctly located more patterns at the first attempt (*c*) of the eight-pattern stage on the PAL task than the treatment as usual (TAU) group. The CT group indicated that they enjoyed playing the game and were motivated to continue across all hours of cognitive training (all ratings higher than 65%) (*d*).

Twenty-two participants (11 male; 11 female) with a diagnosis of schizophrenia were randomly assigned to either the CT (*n* = 10) or TAU (*n* = 12) group and completed all tasks and questionnaires required of their condition. These groups showed no significant differences in intelligence, age, years of education and psychiatric symptoms (including negative symptoms) at baseline assessment (table 1). When analysing performance on the CANTAB PAL, we focused on the eight-pattern stage, the highest level of task difficulty (table 2). At outcome assessment, the CT group made significantly fewer errors and needed significantly fewer attempts to remember the location of different patterns (trial score) relative to the TAU group. The CT group also correctly located more patterns at the first attempt of the eight-pattern stage at outcome assessment. Furthermore, mean GAF scores improved significantly in the CT group from baseline to outcome assessment (table 2). When conducting ANCOVAs including covariates such as baseline measures, the pattern of results did not substantially change. During training, participants' levels of enjoyment and motivation to continue playing were monitored using visual analogue scales on an hourly basis. Participants in the CT group indicated that they enjoyed the game and were motivated to continue playing across the 8 h of CT (all ratings were higher than 65% across all hours of play; figure 1*d*). One-sample *t*-tests comparing participants' average enjoyment and motivation to continue playing across the 8 h demonstrated significantly higher means than 50% (i.e. the level of indifference on the visual analogue scales) for enjoyment (*t*(9) = 15.12, *p* < 0.001, mean enjoyment level = 71.59, s.d. = 4.03) and motivation (*t*(9) = 15.38, *p* < 0.001, mean motivation level = 72.32, s.d. = 4.10). Correlational analyses further demonstrated that iPad peak performance (i.e. the highest level attained out of a total of 20 levels on our memory game across 8 h of training) was positively associated with level of motivation (*p* = 0.03, Spearman's *R* = 0.68), confirming that high levels of performance were associated with task-related (i.e. game play) motivation.

**Table 1.** Demographic data (baseline). Independent samples *t*-test, *χ*^2^-square test. For CT group and TAU group, data shown are means, with standard deviations in parentheses.

measure	CT group	TAU group	statistics
*N*	10	12	
gender	5 M: 5 F	6 M: 6 F	
age	28.70 (6.89)	28.3 (9.15)	*t*(20) = 0.10, *p* = 0.92
years of education	13.90 (3.93)	15.42 (3.55)	*t*(20) = −0.95, *p* = 0.35
NART	104.10 (16.57)	102.25 (10.41)	*t*(20) = 0.32, *p* = 0.75
BPRS	54.90 (8.88)	52.75 (15.45)	*t*(20) = 0.41, *p* = 0.69
NEGATIVE symptoms	2.53 (1.04)	2.69 (1.32)	*t*(20) = −0.04, *p* = 0.97
chlorpromazine equivalents (mg)	385.00	364.00	*t*(15) = 0.16, *p* = 0.87
unmedicated	1	4	*χ*^2^-square = 1.69, *p* = 0.19

**Table 2.** Performance on the CANTAB PAL and GAF scores at baseline and outcome assessments. The baseline and outcome data shown are means, with standard deviations in parentheses. PAL, paired associates learning task.

PAL measure	baseline	statistics	outcome	statistics
errors	CT: 9.33 (9.47) TAU: 13.55 (13.44)	*t*(18) = −1.02, *p* = 0.32	CT: 3.00 (4.61) TAU: 10.27 (10.56)	*t*(18) = −2.38, *p* = 0.03^a^
trials	CT: 2.90 (2.02) TAU: 5.00 (2.86)	*t*(18.04) = −2.04, *p* = 0.06	CT: 1.90 (1.19) TAU: 4.5 (3.05)	*t*(19.98) = −2.83, *p* = 0.01^a^
first trial memory score	CT: 29.50 (18.56) TAU: 17.81 (4.53)	*t*(9.87) = 1.93, *p* = 0.08	CT: 45.00 (23.91) TAU: 22.72 (9.82)	*t*(11.72) = 2.74, *p* = 0.02
GAF	CT: 64.10 (24.88) TAU: 65.42 (26.80)	*t*(20) = −0.12, *p* = 0.91	CT: 72.00 (24.95)^b^ TAU: 64.91 (22.15)	*t*(20) = −2.19, *p* = 0.04^c^

^a^Independent samples *t*-tests were performed on square root transformed data owing to a non-normal distribution.

^b^A paired-samples *t*-test indicated that mean GAF scores significantly improved within the CT group, *t*(9) = −3.30, *p* = 0.009.

^c^Independent samples *t*-test of the changes in GAF scores in the CT group.

As predicted, we found that individuals who completed 8 h of gameplay showed improved performance on the PAL task in terms of reduced errors, improved trial score and the correct number of patterns located (table 2 and figure 1*a–c*). Although further studies with larger sample sizes are required to confirm the clinical generalizability of our current findings (including the potential for transfer effects on other cognitive domains), our data suggest that those who completed training were able to achieve better performance as the difficulty of the task increased. It is possible that use of a computer game (rather than standard cognitive testing using a computer) helped maintain participants' motivation to perform well at a higher level of task difficulty. Non-significant group differences in the BPRS at baseline confirm that the effects of CT could not be attributed to baseline differences in psychiatric symptoms. Given the repetitive nature of most CT programmes, it is plausible that participants' level of enjoyment might decrease over time. In contrast, enjoyment levels associated with playing computer games might increase as players reach more difficult levels and can ‘unlock’ more of the game's potential. We observed that participants' enjoyment and motivation to continue playing were maintained throughout all hours of training, suggesting that our memory game had the same motivational engagement associated with typical computer games.

Enhanced performance at a higher level of the PAL task may represent strengthened competency in the specific network underlying episodic memory, yet importantly, we also found improved functional outcome in the CT group as measured by the GAF at outcome assessment. This suggests that the effectiveness of our memory game might not be limited to improved episodic memory, but it also has the potential to improve functioning in activities of daily living. This functional improvement could be driven by several possible mechanisms. For example, improvements in memory may have had a direct impact on global functions. An alternative explanation is that CT may have had an indirect impact on functionality by improving low self-esteem. Or indeed, both of these explanations may have played a role in terms of the impact of training on functional outcome. The main limitations of this study are the lack of an active comparison group, such as iPad use without a training game, and the relatively small sample size. Nevertheless, the patients were relatively severely ill and well matched such that they are still representative of patients suffering from schizophrenia.

Further research with larger sample sizes and active control conditions is required to elucidate the psychological mechanisms underpinning the global functioning improvements observed here. These results add to a growing body of literature that attests to the efficacy of CT programmes in schizophrenia. Despite support for its therapeutic benefits, CT remains an underused therapeutic option. Potential explanatory factors for this include the long periods of supervised training that are typically required, the need to attend a clinic or hospital setting and the lack of enjoyable, attention-grabbing tasks. This study overcame these limitations through development of a portable hand-held memory game that would be fun for patients to play and concurrently have the potential to provide therapeutic benefits. Here, the iPad game was the result of a 9-month collaboration between psychologists, neuroscientists, a professional game-developer and service users. To the best of our knowledge, this is the first study to have developed a neurotechnology using feedback directly from individuals with schizophrenia to ensure that the narrative was understandable and that playing the game was highly enjoyable. We also used a neurobiological approach to CT based on previous neuropsychological and neuroimaging published findings. These data suggest that use of neurotechnologies may serve to complement current psychological therapies and/or psychopharmacological treatments for schizophrenia. Neurotechnologies may also have potential as treatment applications in other groups, such as healthy elderly individuals or patients groups with memory-related difficulties (e.g. mild cognitive impairment; traumatic brain injury). Using modern technology in mental health may be one method for improving patients' active engagement in treatments [5].

### (f) Summary: cognitive enhancers in neuropsychiatric disorders

In summary, there is a demand for improving and developing treatments for cognitive deficits in neuropsychiatric disorders. In some disorders, such as ADHD and Alzheimer's disease, these symptoms are more obvious and research has focused on treating them, resulting in a sufficient basis for clinical decisions. However, in many other neuropsychiatric disorders, cognitive symptoms have not been fully defined, nor have they been regarded as targets for treatment. Therefore, studies on the function and dysfunction of cognitive domains in neuropsychiatric disorders and their manipulation by pharmacological agents in healthy states and in neuropsychiatric disorders as well as the development of novel cognition-modifying drugs are necessary. In addition, it is important to harness the potential of novel devices and technologies for improving functioning, quality of life and wellbeing in healthcare in general, and in particular in neuropsychiatric disorders. Although experts realize the potential of these inventions and technologies in the treatment of disorders, we have not yet capitalized on them to improve brain health [5].

Furthermore, studies on the combination of different treatment strategies, including for example, combinations of cognitive enhancing drugs and/or CT with anti-psychotics, antidepressants, psychotherapy and the optimal time point in the course of a disorder (acute episode versus early remission versus relapse prevention), are urgently needed.

From an ethical perspective, there can be few doubts about research, development and use of cognition-enhancing drugs in neuropsychiatric disorders, as in these disorders the suffering of the affected individuals, their families and the burden for society are clearly significant and any successful treatment will have widely accepted benefits. In a clinical setting, the neuropsychiatric patients will be monitored by a healthcare professional in regard to drug–drug interactions, counterindications and side effects. Therefore, the evidence base on safety will increase with increasing use, such that in future, benefits and risk can be better evaluated.

In §2, we discuss the increasing lifestyle use of cognitive enhancers by healthy people.

## 2. Cognitive enhancers in healthy people

The drugs used in patients are all prescription drugs. From a legal perspective, amphetamine and methylphenidate are classified as schedule II drugs, and therefore not legally obtainable without medical prescription. The reason for these limitations is the abuse potential of these drugs. Owing to the lower abuse risk, modafinil is a prescription-only drug, but not scheduled in countries such as UK, USA, Canada, Germany and Australia. However, owing to their performance-modulating effects, all these drugs are banned as doping substances in sports competitions.

### (a) Effects of amphetamine, methylphenidate and modafinil in healthy people

The Care Quality Commission reported that over a six year period from 2007 to 2013, there had been a 56% rise in prescriptions for methylphenidate in the UK. They attributed this increase to its use in the management of childhood and adult ADHD, but importantly, also, to its potential for diversion and misuse [1]. Recent reviews summarizing the effects of amphetamine and methylphenidate on cognitive performance in healthy participants showed a couple of consistent effects in the following domains [110,111].

Learning is positively affected by both drugs, but primarily when testing delayed recall and recognition, pointing to an effect on memory consolidation [110]. When testing the effects on prefrontal functions such as attention, working memory, inhibition and planning, the biggest effects were found in participants with lower than optimal performance [65]. In studies of inhibitory control in healthy volunteers, positive effects were found with modafinil [112]. In working memory tasks, the findings were more mixed, with some studies with modafinil finding improvements in working memory with reduced errors or reduced reaction time [113] and others reporting no effect. The reason for these mixed findings might also be influenced by baseline effects (reduced effect in high-performing participants). For example, Muller *et al.* [113] used a difficult version of the CANTAB spatial working memory test, thus avoiding ceiling effects, and found that modafinil improved the performance of healthy volunteers. Inhibitory cognitive control processes are significantly improved by amphetamine and methylphenidate. Other executive functions such as planning (Tower of London, strategic choice task, sequence forming) and fluency have also been investigated. Planning performance on the Tower of London test was improved with modafinil [100]. All these effects were investigated with single doses. Only a few studies investigated the effects of repeated intakes [111], reporting primarily subjective feelings of increased energy and wakefulness. In the few studies in sleep-deprived individuals, methylphenidate seemed primarily to improve subjective feelings of energy and wakefulness, but had relatively small effects on cognitive performance. However, modafinil improved working memory, planning, decision making and flexibility in sleep-deprived doctors [114] without showing the typical side effects of caffeine, such as tremor and anxiety [115]. In the case of atomoxetine, research into cognitive effects is still ongoing. The most consistent finding is an improvement in response inhibition [116,117].

### (b) Non-pharmacological cognitive enhancement in healthy people

Non-pharmacological methods, such as tDCS and TMS, have been investigated regarding their cognition modulating and enhancing functions. These methods apply magnetic fields (TMS) or electrical currents (tDCS) to influence either the activity and/or excitability of certain brain areas. Specifically, tDCS is thought to have rather lasting, neuroplastic effects [118]. One recent review [119] summarized studies applying TMS in patients with neuropsychiatric disorders, such as depression. Verbal learning improved in two studies [120,121], as did executive function [122,123]. However, 8 of 13 studies found no effect on cognition. One of five studies found positive effects on verbal learning in patients with schizophrenia [124], and in patients with Alzheimer's disease, there were two positive studies. However, the few studies that exist are unfortunately underpowered and only some are sham-controlled. Therefore, this field requires more rigorous research on stimulation location, dosage and target symptoms. While the evidence of an antidepressant effect of TMS is established (Hedge's *g* = 0.55 in a meta-analysis [125]) and indeed approved for treatment-resistant depression by the Food and Drug Administration, the evidence for clinical therapeutic effects in other neuropsychiatric disorders and also the effects on specific cognitive functions are still not very strong, requiring more research. The safety profile of TMS is well characterized [126,127]. TDCS, however, has until now been much less investigated in treatment studies (e.g. meta-analyses in depression based on six to seven studies [128,129], only three double-blind placebo-controlled studies on tDCS in tinnitus [118]), and only a few studies have examined the effects of tDCS on cognition in neuropsychiatric disorders (e.g. improved working memory and verbal fluency in schizophrenia [130]). The number of studies on cognitive effects in healthy volunteers is somewhat larger, and they point to improvements in a range of cognitive functions, including memory and attention [131,132]. For instance, a recent study compared the effect of prefrontal tDCS in sleep-deprived healthy volunteers with those of caffeine and found similar to even stronger improvements of vigilance/sustained attention and working memory [133]. However, tDCS requires further research on safety and long-term effects [134,135], particularly when considering the uncontrolled purchasing over the internet and uncontrolled use of these devices [136].

## 3. Summary and conclusion

In summary, these studies suggest that cognitive enhancers such as smart drugs and devices have the potential to provide benefits in healthy people, although the extent and specificity (which intervention affects which function and is best applied in which situation) are still under investigation. Other questions such as the safety of regular use in healthy people and, even more importantly, the potential effects on still-developing brains when used in healthy children and adolescents require more research [5,135,137].

In addition to safety issues, there are also ethical concerns. The exact numbers of healthy users of prescription drugs with the intention to enhance cognition are unknown, with estimates in the general population of less than 5%, although in some studies in students, between 10% and 20% of the students reported to have used prescription drugs within the past year, without strong differences between the US and Europe [110,138–142]. One study in a small competitive college reported use within the last year in more than 30% of the students [143]. However, the prescription rates of stimulants in England have been rising steadily from 220 000 in 1998 to 418 300 in 2004. The global market share of modafinil, licensed in the UK for narcolepsy and excessive fatigue and sleepiness in certain chronic medical conditions, was more than $700 million per year [144], with an estimated ‘off-label’ use of around 90% [145]. In the lay press, modafinil has also gained a lot of attention as a pill that can boost brain power, which reflects the interest of the public in pharmacological enhancement of cognitive abilities such as planning and problem solving. These numbers raise the question as to why healthy people are using cognitive enhancing drugs. Some studies in college students in the USA have asked this question, and most students gave ‘improving intellectual performance’, ‘being more efficient on academic assignments', ‘improving concentration’ and ‘being able to study longer’ as motives, with only a few reporting recreational ones [110]. This matches a report of the Academy of Medical Sciences that a small percentage increment in performance can lead to significant improvements in functional outcome; it is conceivable that a 10% improvement in memory score could lead to an improvement in an A-level grade or degree class [146]. Therefore, many students are using cognitive enhancing drugs to ‘get the competitive edge’ over other students in exam situations. It might have been this consideration that made Duke University prohibit the use of prescription drugs by students without an authorized prescription. Duke University's website specifies the ‘unauthorized use of prescription medication to enhance academic performance’ as cheating in the category ‘academic dishonesty’ of their academic conduct policy [147].

Furthermore, particularly modafinil seems to affect motivation in a manner that makes unappealing tasks more appealing and therefore they can be undertaken and completed more easily. In other words, overall task-related pleasure is increased by modafinil [113]. This increase in motivation is task-specific, and not a general stimulating effect [113]. A third reason reported is reducing the effects of jet-lag and staying awake and alert for longer periods of time.

## 4. Further considerations

As responsible scientists and clinicians in the field of neuroscience and mental health, we need to consider how our discoveries and inventions will affect society [148–150]. In the case of pharmacological cognitive enhancement, there are many people with neuropsychiatric disorders who could benefit greatly from these drugs. In the development of the field of novel pharmacological cognitive enhancers, we must gain maximum benefits with minimum harm to the individual and to society as a whole. However, ethical considerations of cognitive enhancement in healthy people are more complex, ranging from potential benefits in surgeons, shift workers, military, air traffic controllers and other fields where constant high performance is essential, to fears of coercion [151] and competitive pressure by peers, as well as increasing inequality with access depending on wealth and also cheating (see above, academic dishonesty). These ‘smart drugs' may alter the future of work as we know it, particularly where shift work, jet lag and high-risk decisions may compromise performance [152]. On the other hand, as detailed above, scientific knowledge about the specific effects and side effects in the healthy population, particularly with long-term use, is still limited. Therefore, we need collaboration between scientists, pharmaceutical companies and governmental regulators to develop improved substances, get them trialled, tested and licensed. With this knowledge, neuroscientists together with social scientists, philosophers, ethicists and society should actively discuss the ethical and moral consequences of cognitive enhancement.
